# Microglia influence host defense, disease, and repair following murine coronavirus infection of the central nervous system

**DOI:** 10.1002/glia.23844

**Published:** 2020-05-25

**Authors:** Vrushali Mangale, Amber R. Syage, H. Atakan Ekiz, Dominic D. Skinner, Yuting Cheng, Colleen L. Stone, R. Marshall Brown, Ryan M. O'Connell, Kim N. Green, Thomas E. Lane

**Affiliations:** ^1^ Division of Microbiology & Immunology, Department of Pathology University of Utah School of Medicine Salt Lake City Utah USA; ^2^ Department of Neurobiology & Behavior, School of Biological Sciences University of California Irvine California USA

**Keywords:** coronavirus, demyelination, host defense, microglia, remyelination

## Abstract

The present study examines functional contributions of microglia in host defense, demyelination, and remyelination following infection of susceptible mice with a neurotropic coronavirus. Treatment with PLX5622, an inhibitor of colony stimulating factor 1 receptor (CSF1R) that efficiently depletes microglia, prior to infection of the central nervous system (CNS) with the neurotropic JHM strain of mouse hepatitis virus (JHMV) resulted in increased mortality compared with control mice that correlated with impaired control of viral replication. Single cell RNA sequencing (scRNASeq) of CD45+ cells isolated from the CNS revealed that PLX5622 treatment resulted in muted CD4+ T cell activation profile that was associated with decreased expression of transcripts encoding MHC class II and CD86 in macrophages but not dendritic cells. Evaluation of spinal cord demyelination revealed a marked increase in white matter damage in PLX5622‐treated mice that corresponded with elevated expression of transcripts encoding disease‐associated proteins Osteopontin (*Spp1*), Apolipoprotein E (*Apoe*), and Triggering receptor expressed on myeloid cells 2 (*Trem2*) that were enriched within macrophages. In addition, PLX5622 treatment dampened expression of Cystatin F (*Cst7*), Insulin growth factor 1 (*Igf1*), and lipoprotein lipase (*Lpl*) within macrophage populations which have been implicated in promoting repair of damaged nerve tissue and this was associated with impaired remyelination. Collectively, these findings argue that microglia tailor the CNS microenvironment to enhance control of coronavirus replication as well as dampen the severity of demyelination and influence repair.

## Main points

- Microglia augment host defense in response to CNS infection by the neurotropic coronavirus JHMV.
- A role for microglia in restricting demyelination and promoting remyelination in JHMV infected mice also is demonstrated.

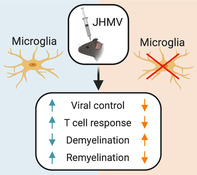

## 1 INTRODUCTION

Intracranial inoculation of C57BL/6 mice with the neurotropic JHM strain of mouse hepatitis virus (JHMV), a member of the *Coronaviridae* family, leads to an acute encephalomyelitis in which virus infects and replicates within glial cells with relative sparing of neurons (Bergmann, Lane, & Stohlman, 2006; Lane & Hosking, 2010; Templeton & Perlman, 2007; Weiss & Leibowitz, 2011). Expression of type I interferon (IFN‐I) is critical in helping control viral replication as mice lacking IFN‐I receptor exhibit increased mortality associated with enhanced viral replication (Ireland, Stohlman, Hinton, Atkinson, & Bergmann, 2008). In addition, localized expression of T cell chemotactic chemokines including CCL5, CXCL9, and CXCL10 within the CNS contribute to host defense by attracting virus‐specific CD4+ and CD8+ T cells into the CNS that further control viral replication through secretion of interferon‐γ (IFN‐γ) and cytolytic activity (Bergmann et al., 2004; Glass et al., 2004; Glass & Lane, 2003a; Glass & Lane, 2003b; Liu et al., 2000; Liu, Armstrong, Hamilton, & Lane, 2001; Marten, Stohlman, & Bergmann, 2001; Parra et al., 1999). Antibody‐secreting cells (ASCs) are also capable of responding to CXCL9 and CXCL10 and aid in host defense (Phares, Marques, Stohlman, Hinton, & Bergmann, 2011; Phares, Stohlman, Hinton, & Bergmann, 2013). Nonetheless, sterile immunity is not achieved and the majority of animals that survive the acute stage of disease develop immune‐mediated demyelination in which both virus‐specific T cells and macrophages amplify the severity of white matter damage associated with hind‐limb paralysis (Bergmann et al., 2006; Hosking & Lane, 2009; Hosking & Lane, 2010; Templeton & Perlman, 2007).

While the functional roles of T cells and B cells in both host defense and disease in JHMV‐infected mice have been extensively studied, there is increasing interest in better understanding how resident cells of the CNS contribute to these events. Microglia are considered the resident immune cells of the CNS and aid in a diverse array of functions including maintaining CNS homeostasis as well as contributing to various disease‐associated conditions (Hammond, Robinton, & Stevens, 2018; Salter & Stevens, 2017; Tejera & Heneka, 2019; Wolf, Boddeke, & Kettenmann, 2017). Moreover, microglia are immunologically competent and capable of rapidly responding to infection and/or damage via specific expression of surface receptors culminating in morphologic changes accompanied by secretion of proinflammatory cytokines/chemokines that function in amplifying neuroinflammation. Recently, the functional role of microglia in contributing to host defense in response to CNS infection with neurotropic viruses has been examined. These studies have been greatly aided by findings demonstrating that mice lacking colony stimulating factor 1 receptor (CSF1R−/−) lack microglia emphasizing the importance of this signaling pathway in microglia development (Ginhoux et al., 2010). Subsequent studies by Green and colleagues (Elmore et al., 2014) showed that blocking CSF1R signaling in adult mice through administration of CSF1R antagonists is also important in survival of microglia in adult mice. Recent studies have employed treatment of mice with PLX5622, a brain penetrant and selective antagonist of the CSF1R that results in a dramatic reduction in microglia, to better understand functional roles of these cells in preclinical models of neurodegenerative disease (Acharya et al., 2016; Dagher et al., 2015; Elmore et al., 2014; Spangenberg et al., 2019). In addition, PLX5622‐mediated targeting of microglia results in increased susceptibility to West Nile virus (WNV) (Funk & Klein, 2019; Seitz, Clarke, & Tyler, 2018), Japanese encephalitis virus (JEV) (Seitz et al., 2018), Theiler's murine encephalomyelitis virus (TMEV) (Sanchez et al., 2019a; Waltl et al., 2018), and JHMV (Wheeler, Sariol, Meyerholz, & Perlman, 2018) arguing for a protective role for microglia against acute viral‐induced encephalitis.

The current study was undertaken to evaluate how microglia tailor the immunological landscape in response to JHMV infection within the brain and spinal cord at different stages of infection with regard to pathways associated with both host defense and neuropathology. We believe microglia will be critical in aiding in host defense through regulating a number of different pathways including antigen presentation and T cell activation as well as augmenting demyelination. To address this, we used a comprehensive set of analytical approaches including single cell RNA sequencing (scRNAseq), flow cytometry, and histopathological techniques to assess disease outcome in JHMV‐infected mice treated with PLX5622 at defined times postinfection. Our findings emphasize an important role for microglia in aiding in host defense in response to JHMV infection of the CNS as well as influencing both the severity of spinal cord demyelination and remyelination in a model of murine coronavirus‐induced neurologic disease.

## 2 MATERIALS AND METHODS

### 2.1 Mice and viral infection

Five‐week‐old C57BL/6 male mice were purchased from The Jackson Laboratory. Mice were infected intracranially (i.c.) with 250 plaque forming units (PFU) of JHMV strain J2.2v‐1 in 30 μl of sterile Hanks balanced sterile solution (HBSS) and animals were euthanized at days 3, 7, 12, and 21 postinfection (p.i.). Clinical disease in JHMV‐infected mice was evaluated using a previously described scale (Lane et al., 2000). To determine viral titers within brains, experimental animals were sacrificed at defined times p.i., brains isolated, homogenized and plaque assay were performed on the DBT astrocytoma cell line as described previously (Hirano, Murakami, Fujiwara, & Matsumoto, 1978). All animal studies were reviewed and approved by the University of Utah Animal Care and Use Committee.

### 2.2 PLX5622 treatment

AIN‐76A (Research Diets, NJ) rodent chow formulated with CSF1R inhibitor‐PLX5622 at a dose of 1,200 mg/kg of chow was kindly provided by Plexxikon, Inc (Berkeley, CA). Mice were fed with either PLX5622 chow or control chow 7 days prior to viral infection and chow was continued until the mice were sacrificed to harvest tissues at defined times p.i.

### 2.3 Cell isolation and flow cytometry

Flow cytometry was performed to identify inflammatory cells entering the CNS using established protocols (Blanc, Rosen, & Lane, 2014; Chen et al., 2014). In brief, single cell suspensions were generated from tissue samples by grinding with frosted microscope slides. Immune cells were enriched via a two‐step Percoll cushion (90 and 63%) and cells were collected at the interface of the two Percoll layers. Before staining with fluorescent antibodies, isolated cells were incubated with anti‐CD16/32 Fc block (BD Biosciences, San Jose, CA) at a 1:200 dilution. Immunophenotyping was performed using commercially available antibodies specific for the following cell surface markers: CD4, CD8, CD11b (BD Biosciences, San Jose, CA), and CD45 (eBioscience, San Diego, CA). The following flow cytometric gating strategies were employed for inflammatory cells isolated from the CNS: macrophages (CD45^hi^CD11b+) and microglia (CD45^lo^ CD11b+). APC‐conjugated rat anti‐mouse CD4 and a PE‐conjugated tetramer specific for the CD4 immunodominant epitope present within the JHMV matrix (M) glycoprotein spanning amino acids 133–147 (M133‐147 tetramer) to determine total and virus‐specific CD4^+^ cells, respectively (Chen et al., 2014; Marro, Grist, & Lane, 2016a); APC‐conjugated rat anti‐mouse CD8a and a PE‐conjugated tetramer specific for the CD8 immunodominant epitope present in the spike (S) glycoprotein spanning amino acids 510‐518 (S510‐518) to identify total and virus‐specific CD8^+^ cells, respectively (Chen et al., 2014; Marro et al., 2016a). Data were collected using a BD LSR Fortessa X‐20 flow cytometer and analyzed with FlowJo software (Tree Star Inc.).

### 2.4 scRNASeq

Immune cells were isolated as described above from brain (day 7 p.i.) and spinal cord (day 14 p.i.) and stained with DAPI and APC conjugated anti‐CD45 for 20 min on ice in 1× PBS containing 0.5% bovine serum albumin (BSA). Live CD45+ cells were enriched through the use of BD FACS Aria flow sorter (University of Utah Health Science Center) and washed once with 0.04% BSA. Samples were then processed for single cell RNA sequencing via the 10× Genomics platform performed at the Huntsman Cancer Institute High Throughput Genomics Shared Resource Core Facility (https://uofuhealth.utah.edu/huntsman/ shared‐resources/gba/). RNA sequencing was performed via Agilent Hiseq next generation sequencer. Sequencing data was processed using the 10X Genomics Cell Ranger pipeline and analyzed using the Seurat R package. Gene expression signatures defining cell clusters were analyzed from PLX5622‐treated and controls at day 7 p.i. (brains) and day 14 p.i. (spinal cords). Cells from each aggregated sample data set were clustered into corresponding immune cell populations by a shared nearest neighbor modularity optimization‐based clustering algorithm using the Seurat package. The resulting clusters were defined using an immune‐cell scoring algorithm (https://aekiz.shinyapps.io/CIPR/) (Ekiz et al., 2019) that compares the gene signatures of each cluster in the experimental data set with the microarray data available in the Immunological Genome (ImmGen) Project Database. Expression levels and distribution of population‐specific immune cell markers were then analyzed to further refine the identified clusters and expose any subpopulations that should be separated as independent clusters. Once the clusters were established and identified, plots were generated using Seurat, ggpubr, and fgsea R packages.

### 2.5 Histology

Mice were euthanized at defined times points according to IACUC‐approved guidelines and the length of spinal cord extending from thoracic vertebrate 6–10 was cryoprotected in 30% sucrose, cut into 1‐mm transverse blocks and processed to preserve the craniocaudal orientation and subsequently embedded in O.C.T. (VWR, Radnor, PA). Eight micron (μm)‐thick coronal sections were cut and sections were stained with hematoxylin/eosin (H&E) in combination with luxol fast blue (LFB) and between 4 and 8 sections/mouse analyzed. Areas of total white matter and demyelinated white matter were determined with ImageJ Software and demyelination was scored as a percentage of total demyelination from spinal cord sections analyzed (Blanc et al., 2015; Blanc, et al., 2014; Dickey, Worne, Glover, Lane, & O'Connell, 2016; Marro, Grist, & Lane, 2016b).

### 2.6 Electron microscopy and *g*‐ratio analysis

For electron microscopy (EM) analysis of spinal cords, mice were sacrificed and underwent cardiac perfusion with 0.1 M cacodylate buffer containing 2% paraformaldehyde/2% glutaraldehyde. Serial ultrathin sections of spinal cords embedded in Epon epoxy resin were stained with uranyl acetate‐lead citrate and analyzed as previously described (Liu, Keirstead, & Lane, 2001). Images at ×1200 magnification were analyzed for *g*‐ratio using ImageJ software. In adult animals there is a relationship between axon circumference and myelin sheath thickness (number of lamellae) expressed by the *g*‐ratio (axon diameter/total fiber diameter); in remyelination this relationship changes such that myelin sheaths are abnormally thin for the axons they surround (Smith, Bostock, & Hall, 1982). An abnormally thin myelin sheath, relative to axonal diameter, was used as the criterion for oligodendrocyte remyelination. Absence of a myelin sheath was used as the criterion for demyelination. For most axons, two measurements were conducted with a minimum of 400 axons analyzed per experimental group. In all cases, slides were blinded and read independently by two investigators.

### 2.7 Statistical analysis

GraphPad Prism was used to perform statistical analyses. Data for each experiment is presented as mean ± *SEM*. For flow cytometry analysis unpaired Student's *t* test was used to determine significance and a *p* value of <.05 was considered statistically significant. Wilcoxon test was used for analyzing gene expression in scRNASeq clusters and the resulting *p* values were corrected for multiple comparisons by Holm‐Sidak method and a *p* value of <.05 was considered statistically significant.

## 3 RESULTS

### 3.1 PLX5622 treatment increases susceptibility to JHMV‐induced neurologic disease

**FIGURE 1:**
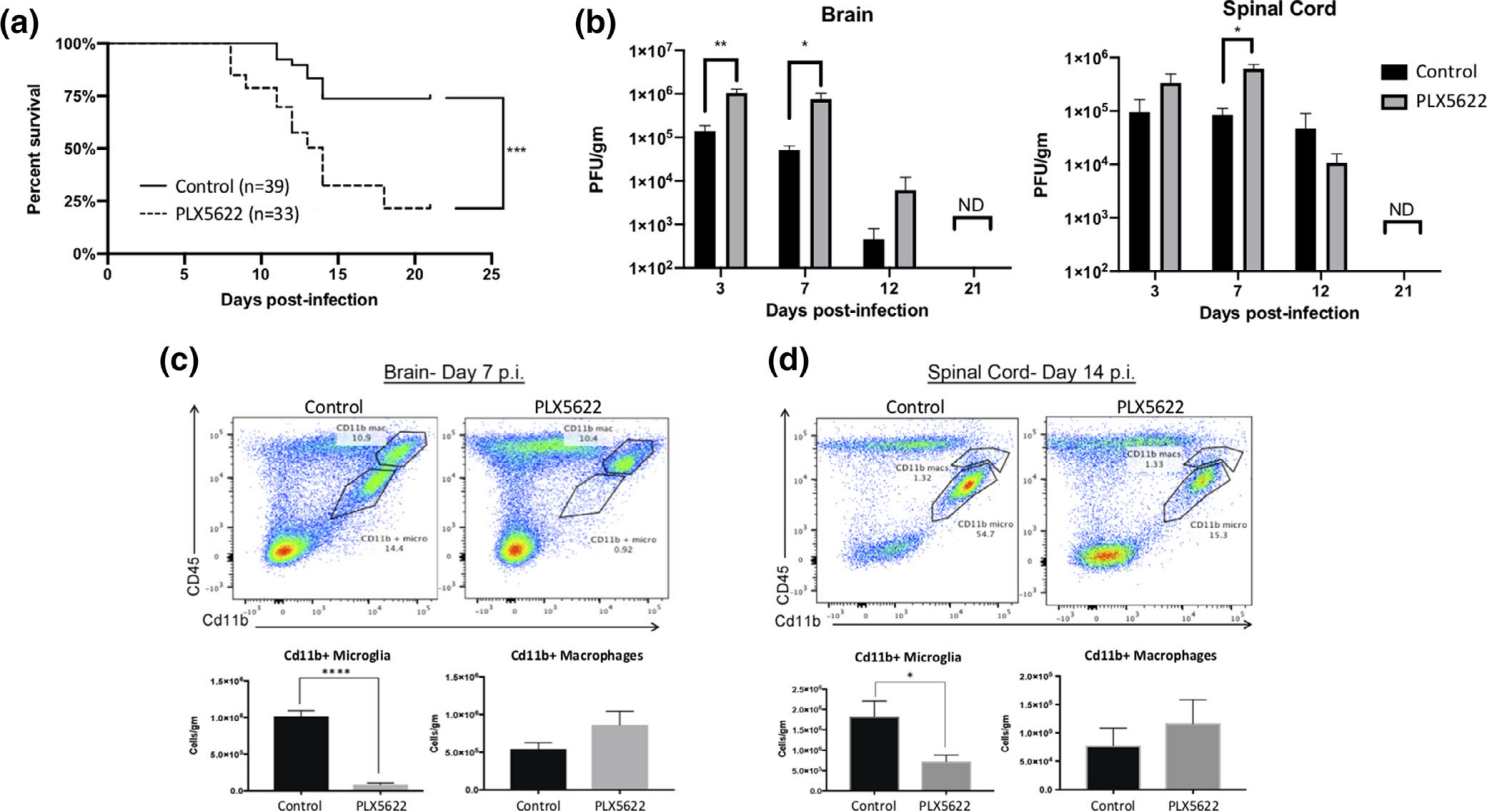
PLX5622 treatment increases susceptibility to JHMV‐induced neurologic disease. Mice were fed either PLX5622 or control chow for 7 days prior to i.c. infection with JHMV (250 PFU) and subsequently remained specific chow for the duration of the experiment. PLX5622 treatment led to (a) increased mortality compared to control mice that was associated with an (b) impaired ability to control viral replication within the brains and spinal cords at days 3, 7, and 12 p.i. compared with control mice. Representative flow cytometric data from JHMV‐infected mice treated with either PLX5622 or control chow and gating on microglia (CD45^lo^CD11b^+^ cells) or macrophages (CD45^hi^ CD11b^+^ cells) in (c) brains at day 7 p.i., and (d) spinal cords at day 14 p.i. PLX5622‐treatment resulted in reduced numbers of microglia in brains and spinal cords compared with control mice. Data are derived from a minimum of three independent experiments with a minimum of 3 mice/time points. Data in B, C, and D are presented as average ± *SEM*. ND, not detected; *p < .05, ****p < .0001

To evaluate the contribution of microglia to disease progression in JHMV‐infected mice, the CSF1R inhibitor PLX5622 was administered as previous studies have reported this pharmacologic approach effectively depletes >90% of microglia (Acharya et al., 2016; Najafi et al., 2018). Mice were treated with PLX5622 (1,200 mg/kg) 7 days prior to infection and continued on the drug for the duration of the experiment. Treatment with PLX5622 resulted in an overall increase in mortality with ~25% of PLX5622‐treated mice surviving to day 21 p.i. whereas ~75% of control‐chow treated mice survived to this time (Figure 1a). The increase in mortality in PLX5622‐treated mice correlated with increased viral titers within the brains and spinal cords at days 3, 7, and 12 p.i. compared with control animals; however, by day 21 p.i. viral titers were not detected (ND) in experimental groups (Figure 1b). We confirmed efficient microglia (CD45^lo^CD11b+) depletion in PLX5622‐treated mice within the brain at day 7 p.i. (Figure 1c) and spinal cord at day 14 p.i. (Figure 1d) using flow cytometry. PLX5622 treatment did not affect numbers of macrophages (CD45^hi^CD11b+) within brains and spinal cords of experimental mice (Figure 1c,d). These findings support earlier work indicating that PLX5622‐targeting of microglia impacts efficient immune‐mediated control of viral replication following infection with neurotropic viruses (Funk & Klein, 2019; Sanchez et al., 2019b; Seitz et al., 2018; Waltl et al., 2018; Wheeler et al., 2018).

### 3.2 PLX5622 treatment and immune cell infiltration into the brains of JHMV‐infected mice during acute disease

**FIGURE 2:**
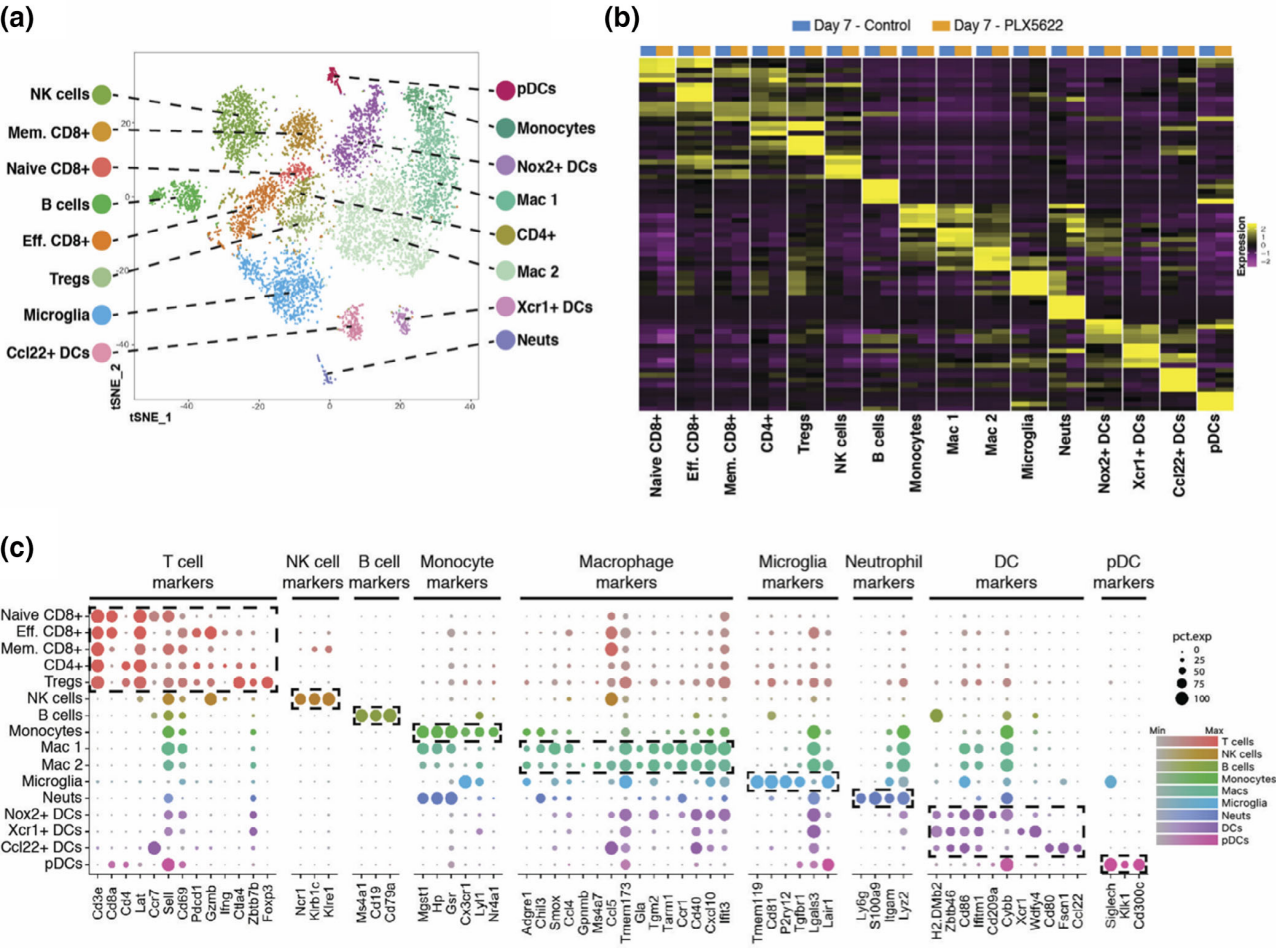
scRNAseq of CD45+ cells isolated from brains of JHMV‐infected mice treated with PLX5622 at day 7 p.i. (a**)** T‐distributed stochastic neighbor embedding (t‐SNE) plot of scRNASeq data revealing 16 distinct cell clusters (aggregate data from PLX5622 and control treated animals at 7 days p.i). (b) Heat map showing the top five differentially expressed genes within each cluster. Columns represent the different clusters, with sub‐columns displaying both control‐treated (blue) and PLX5622‐treated (orange) groups, and rows specify genes. (c) Dot plot presenting expression of selected genes within the 16 cell clusters. Size of the dot is representative of the frequency of cells within a cluster expressing the gene of interest, while the degree of color intensity is indicative of the level of expression of the gene. The dashed boxes highlight commonly and uniquely expressed genes of clusters within overarching cell types

Our findings reveal that PLX5622 treatment of mice increases susceptibility to JHMV‐induced neurologic disease associated with impaired ability to control viral replication. In order to better understand the effects of PLX5622 treatment on influencing the immune cell composition of the CNS we used 10× Genomics scRNAseq technology. Experimental mice were fed either control chow or chow containing PLX5622 for 7 days prior to infection and remained on chow until sacrificed at either day 7 p.i. or 14 p.i., at which point, live CD45+ cells were sorted from the brains or spinal cords, respectively. The respective tissues were found appropriate to study host defense and disease pathogenesis. The immune response to JHMV infection peaks around day 7 p.i. in the brain and ensuing spinal cord demyelination is present at day 14 p.i. We aggregated data from 4,806 cells taken from control‐treated (*n* = 6) and 3,868 cells from PLX5622‐treated (*n* = 6) mice brain tissue at day 7 p.i. and performed unsupervised clustering analysis based on similarity of gene expression signatures using Seurat single cell genomics R package (Ekiz et al., 2019) (Table 1). This approach revealed 16 distinct cell clusters representative of both lymphoid and myeloid linages at day 7 p.i. (Figure 2a). To better understand the overlapping expression of marker genes and identification of cell clusters, we employed a recently described algorithm that compares the gene expression signatures of cell clusters with publicly available ImmGen database (Ekiz et al., 2019). As previously described (Ekiz et al., 2019), this algorithm calculates an aggregate identity score for each scRNAseq cell cluster as a measure of molecular similarity to the ImmGen subsets. Through combinations of these two approaches, we identified three CD8+ T cell subsets [naïve, effector (Eff.), and memory (Mem.)], two macrophage subsets (Mac 1 and Mac 2), four dendritic cell (DC) subsets (plasmacytoid, NADPH [*Nox2*], XCR1 [*Xcr1*], CCL22 [*Ccl22*]), and single subsets of CD4+ T cells, regulatory T cells (Treg), natural killer (NK) cells, B cells, microglia, neutrophils (neuts), and monocytes at day 7 p.i. (Figure 2a). In order to verify the algorithm‐assisted identification of cell clusters, we examined expression of known cellular markers in our data set; expression of these markers corresponded with the respective identities of the distinct clusters (Figure 2b,c). Our initial preliminary analyses focusing on samples separately are in agreement with the results of this aggregated approach.

**TABLE 1:** Overview of experimental conditions showing treatment, sacrifice time points, tissue collected, and total number of CD45+ cells isolated as well as reads/cell following scRNAseq analysis

Time point	Day 7 p.i.	Day 7 p.i.	Day 14 p.i.	Day 14 p.i.
Diet	Control chow	PLX5622	Control chow	PLX5622
Tissue	Brain	Brain	Spinal cord	Spinal cord
Pooled N	6	6	7	5
Cell number	4,806	3,868	2,725	4,891
Reads/cell	54,593	61,984	111,721	41,498

**FIGURE 3:**
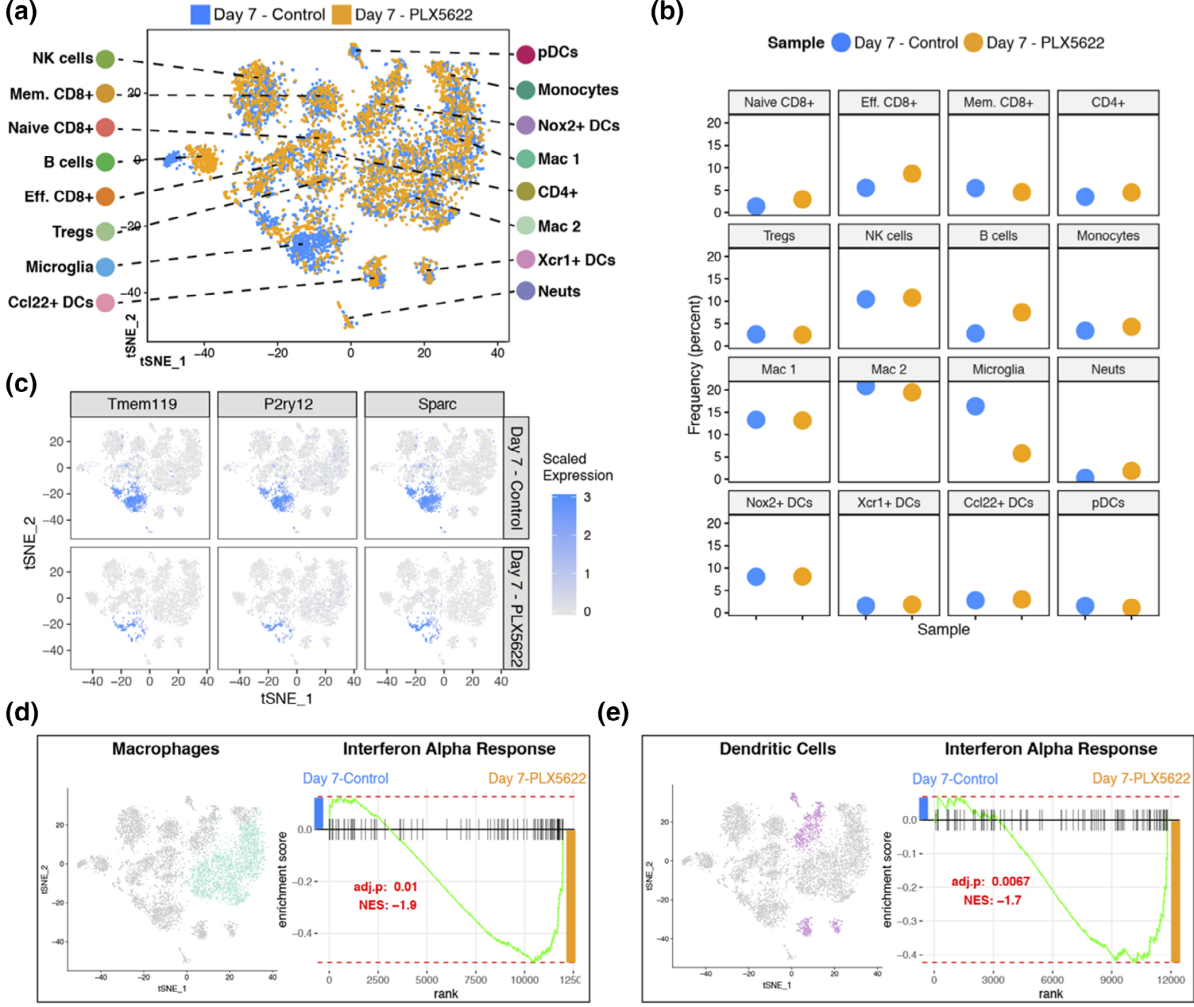
PLX5622 treatment and immune cell infiltration into the CNS at day 7 p.i. (a) t‐SNE plot showing the immune landscape in brains of control‐(blue) and PLX5622‐(orange) treated animals at 7 days p.i. (b) Frequency of cell clusters in brains of control‐ and PLX5622‐treated animals at 7 days post JHMV infection. (c) t‐SNE plot showing decreased expression of microglia‐associated transcripts *Tmem119*, *P2ry12*, and *Sparc* in PLX5622 mice compared with controls. Gene set enrichment analysis (GSEA) for IFN‐α responses in combined (d) macrophage (teal) and (e) dendritic cell (purple) populations isolated at day 7 from brains of JHMV‐infected mice treated with either PLX5622 or control chow, represented in t‐SNE plots. Area under the curve represents enrichment of response genes. Responses to IFN‐α were enriched in both macrophages and dendritic cells isolated from brains of PLX5622 mice compared with control animals at day 7 p.i. Normalized enrichment scores and *p* values are shown

We next analyzed differences in CD45+ cells between PLX5622‐treated and control mice at day 7 p.i. following JHMV infection. When data from the cellular genotypes were plotted side‐by‐side, treatment‐dependent dynamics within the tissues started to emerge (Figure 3a,b). Importantly, we were able to show that PLX5622 treatment resulted in decreased expression of microglia‐associated transcripts *Tmem119*, *P2ry12*, and *Sparc* compared with control mice (Figure 3c). Expression of IFN‐α is critical in effective control of JHMV replication within the CNS (Athmer et al., 2018; Ireland et al., 2008; Vijay et al., 2017). We examined IFN‐α responses by both macrophages and DCs in order to gain better insight into potential mechanisms by which PLX5622 treatment led to impaired control of viral replication. When the global expression signatures of macrophages and DCs were examined, IFN‐α response genes were found to be significantly enriched within the brains of PLX5622‐treated mice compared with controls arguing that targeting microglia did not diminish IFN‐I responses (Figure 3d,e).

### 3.3 PLX5622‐treatment alters infiltration and activation phenotype of T cells during acute disease

Control of JHMV replication within the CNS is associated with infiltration of activated virus‐specific CD4+ and CD8+ T cells (Marten et al., 2001; Pearce, Hobbs, McGraw, & Buchmeier, 1994; Williamson & Stohlman, 1990). At day 7 p.i., PLX5622 treatment did not significantly alter CD4+ T cell infiltration yet there was an increase in CD8+ T cells (*p* < .05) compared with control mice as determined by flow cytometric analysis (Figure S1a). There were no differences in virus‐specific CD4+ and CD8+ T cells specific for immunodominant epitopes present within the Matrix (M) (Figure S1b) and Spike (S) glycoproteins (Figure S1c) as determined by tetramer staining (Blanc, et al., 2014; Marro et al., 2016b).

**FIGURE 4:**
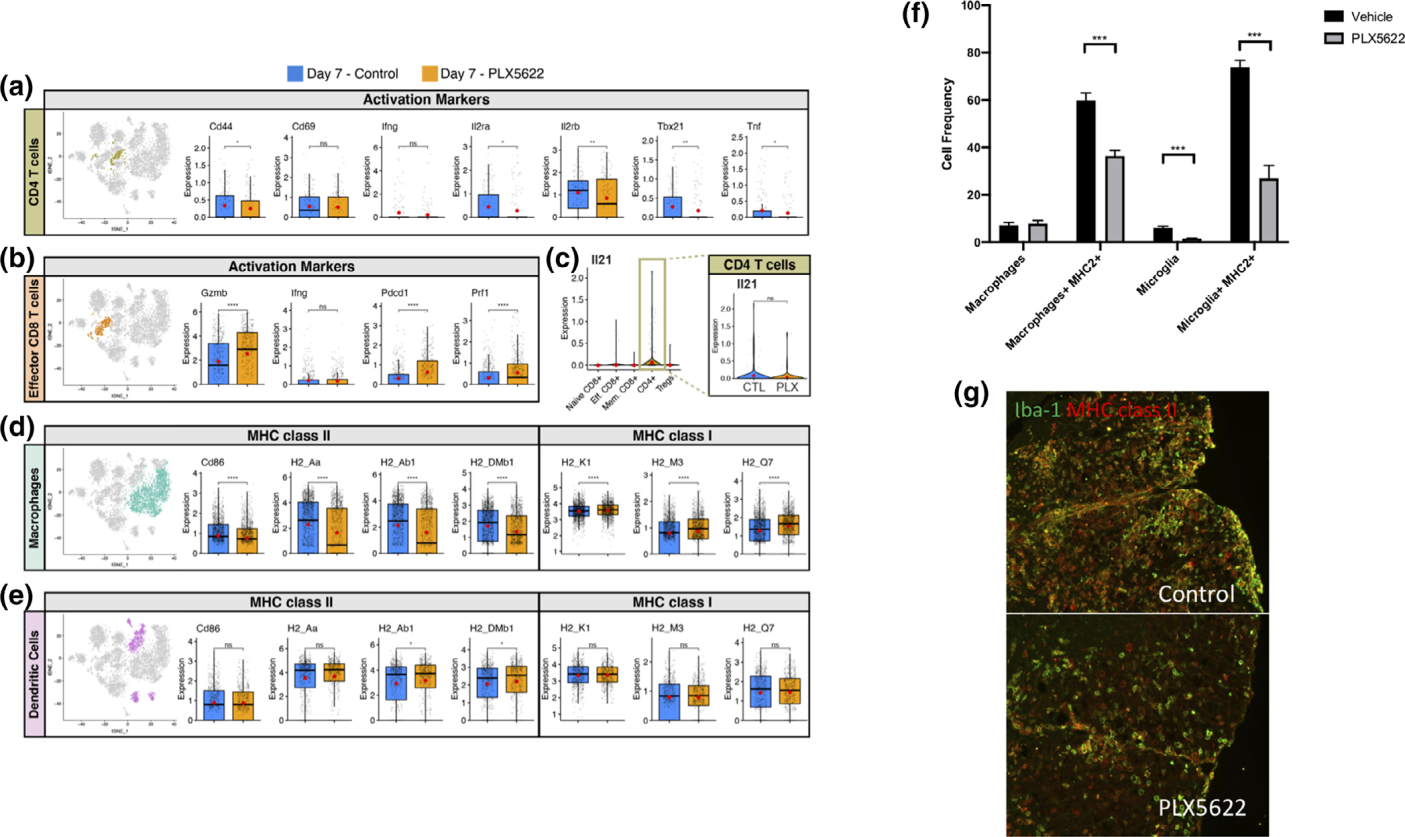
Altered T cell activation profiles in PLX5622‐treated mice at day 7 p.i. t‐SNE plot from brains of JHMV‐infected mice treated with PLX5622 or control at day 7 p.i. showing (a) CD4+ T cells from either PLX5622‐treated or control mice and box plots comparing expression of transcripts encoding for activation markers *Cd44* (CD44), *Cd69* (CD69), *Il2ra* (IL‐2 receptor subunit alpha) *Il2rb* (IL‐2 receptor subunit beta), *Tbx21* (Transcription factor T‐bet) *Ifng* (interferon gamma), and *Tnf* (tumor necrosis factor alpha). (b) CD8+ T cells comparing the expression levels of transcripts encoding effector and activation markers in CD8+ T cells *Prf1* (Perforin), *Pdcd1* (Programmed cell death 1, PD1), *Gzmb* (Granzyme B), and *Ifng*. (c) Violin plots depicting expression of *Il21* transcripts within T cell populations isolated from the brains of experimental mice; there was no significant (ns) difference in expression of *Il21* in CD4+ T cells from control or PLX5622‐treated mice. Expression of MHC class II‐associated genes (*H2‐Aa*, *H2‐Ab1*, and *H‐2DMb1*) and co‐stimulatory molecule *Cd86*, as well as MHC class I‐associated genes (*H2‐K1*, *H2‐M3*, and *H2‐Q7*), are shown in (d) macrophages and (e) dendritic cells from experimental mice. In these plots, each dot represents a single cell. Normalized expression values were used and random noise was added to show the distribution of data points. The box plots show interquartile range and the median value (bold horizontal bar). Average expression value per sample is indicated by the red dots. Wilcoxon's test was used for statistical analysis. (f) MHC class II expression on macrophages (CD11b + CD45^high^) and microglia (CD11b + CD45^lo^) isolated from the brains of JHMV‐infected mice treated with either PLX5622 or control at day 7 p.i. as determined by flow cytometric analysis. Data presented as average + *SEM*. (b) Representative immunofluorescent staining for MHC class II on spinal cords isolated from infected mice treated with either PLX5622 or control at day 7 p.i. ns, not significant; *p < .05; **p < .01 ***p ≤ .001, ****p ≤ .0001

Evaluation of defined factors associated with T cell activation at day 7 p.i. revealed reduced expression of the Th1‐associated transcription factor Tbet (*Tbx21*) (*p* < .01) and this was associated with reduced (*p* < .05) expression of *Tnf* transcripts, but not *Ifng* transcripts, in PLX5622‐treated mice compared to control mice (Figure 4a). We also determined reduced expression of activation markers CD69 (*Cd69*) and CD44 (*Cd44*, *p* < .05) in CD4+ T cells from the brains of PLX5622‐treated mice compared to control mice at day 7 p.i. (Figure 4a). In addition, the CD4+ T cells subset from PLX5622‐treated mice also expressed reduced transcripts for *Il2ra* (*p* < .05)and *Il2rb* (*p* < .01) that encode for components of the IL‐2 receptor when compared to control‐treated mice (Figure 4a). Previous work from Bergmann and colleagues (Phares et al., 2013) identified that IL‐21 derived from CD4+ T cells is important in enhancing anti‐viral effector functions by CD8+ T cells following JHMV infection. Comparison of *Il21* transcript levels in CD4+ T cells between PLX5622‐treated mice compared to control mice indicated no significant difference at day 7 p.i. (Figure 4c). In contrast, CD8+ T cells isolated from brains of PLX5622‐treated mice expressed increased transcripts specific for perforin (*Prf1*, *p* < .0001), granzyme B (*Gzmb*, *p* < .001) and programmed cell death 1, PD‐1 (*Pdcd1*, *p* < .0001) (Figure 4b). There was no difference in expression of *Ifng* transcripts between experimental groups (Figure 4b).

Correlating with muted CD4+ T cell activation was the demonstration of a reduction (*p* < .0001) in transcripts associated with MHC class II as well as the co‐stimulatory molecule CD86 in macrophages in PLX5622‐treated mice compared controls (Figure 4d). Flow cytometric staining for MHC class II on cells isolated from the brains of PLX5622 and control mice at day 7 p.i. confirmed expression was reduced (*p* < .001) on macrophages obtained from PLX5622‐treated mice compared to controls (Figure 4f). Additionally, MHC class II expression on remaining microglia within the brains of PLX5622‐treated mice was also reduced (*p* < .001) compared to expression on microglia from control mice (Figure 4f). Dampened expression of MHC class II in PLX5622‐treated mice was further confirmed through immunofluorescent staining in combination with Iba1 (Figure 4g). Expression of MHC class I‐associated transcripts was increased (*p* < .0001) in macrophages isolated from the brains of PLX5622‐treated mice compared with control animals in macrophages which was consistent with apparent increased CD8+ T cell activation (Figure 4d). With regard to dendritic cells at day 7 p.i., we did not detect changes in expression of transcripts associated with either MHC Class II or MHC Class I (Figure 4e) between PLX5622‐treated mice and controls.

### 3.4 PLX5622 treatment and immune cell infiltration into spinal cords of JHMV‐infected mice during immune‐mediated demyelination

**FIGURE 5:**
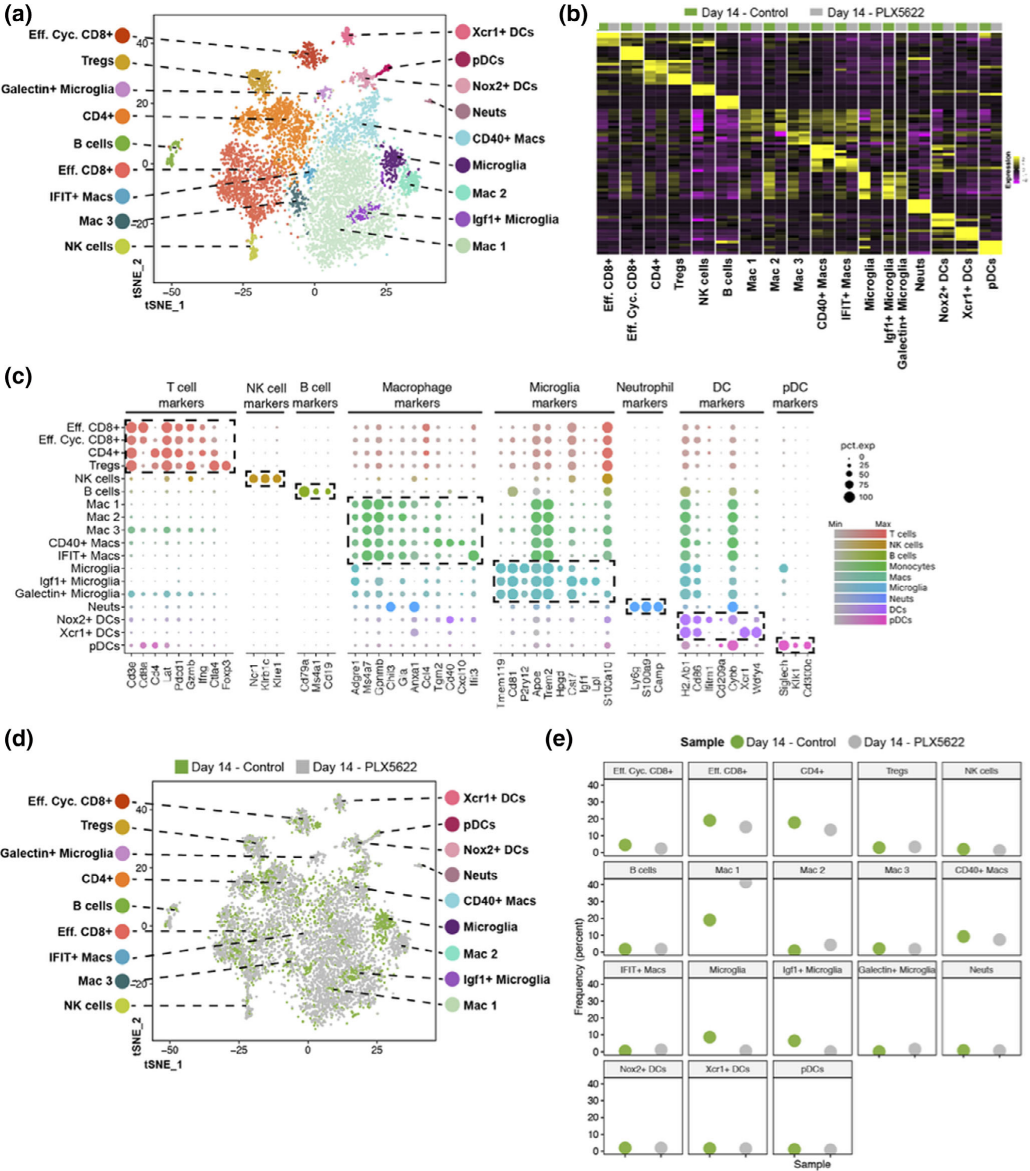
scRNAseq of CD45+ cells isolated from spinal cords of JHMV‐infected mice treated with PLX5622 at day 14 p.i. (a) t‐SNE plots of scRNASeq data revealing 18 distinct cell clusters. Aggregate data are from spinal cords isolated from PLX5622‐ and control‐treated animals at 14 days p.i. (b) Heat map showing the top five differentially expressed genes within each cluster. Columns represent the different clusters, with sub‐columns displaying both control‐treated (green) and PLX5622‐treated (gray) groups, and rows specify genes. (c) Dot plot presenting expression of selected genes within the 18 cell clusters. Size of the dot is representative of the frequency of cells within a cluster expressing the gene of interest, while the degree of color intensity is indicative of the level of expression of the gene. The dashed boxes highlight commonly and uniquely expressed genes of clusters within overarching cell types. (d) t‐SNE plot showing the immune landscape in spinal cords of control‐(green) and PLX5622‐(gray) treated animals at day14 p.i. (e) Frequency of cell clusters in spinal cords of control‐ and PLX5622‐treated animals at day 14 p.i.

We next performed scRNAseq on CD45+ cells enriched from the spinal cords of JHMV‐infected mice treated with either PLX5622 or control at day 14 p.i. Using the same approach as described above using aggregated data from 2,725 cells taken from control‐treated (*n* = 6) and 4,891 cells from PLX5622‐treated (*n* = 6) mice (Table 1), we detected 18 cell clusters within spinal cords of experimental mice representing lymphoid and myeloid populations (Figure 5a). In brief, this included 3 DC subsets (plasmacytoid, NADPH [*Nox2*], XCR1 [*Xcr1*]), five macrophage subsets (CD40 [*Cd40*], [Interferon‐induced proteins with Tetratricopeptide repeats IFIT (*Ifit1*,*Ifit2*,*Ifit3*)], Mac 1, Mac 2, and Mac 3), three populations of microglia that included Galectin+ microglia and Insulin growth factor 1‐positive (*Igf1+*) microglia, two populations of CD8+ T cells [effector (Eff.) and effector cycling (Eff. Cyc.)], and single populations of neuts, Tregs, CD4+ T cells and B cells. Expression of known cellular markers in our data set corresponded with respective identities of clusters (Figure 5b,c). Consistent with our spinal cord flow data (Figure 1d), PLX5622 treatment led to a reduction in microglia with a trend toward an increase in macrophage populations (Figure 5d,e). The presence of *Ifit*‐positive macrophages argues for a role in contributing to anti‐viral immune responses (Diamond & Farzan, 2013). Further, we also detected the presence of Galectin‐positive microglia although there were no differences in frequency between experimental populations (Figure 5d,e). Finally, we did find a reduced frequency of *Igf‐1*‐positive microglia within the spinal cords of PLX5622‐treated mice compared to controls (Figure 5d,e).

### 3.5 CSF1R antagonism increases the severity of demyelination in JHMV‐infected mice

**FIGURE 6:**
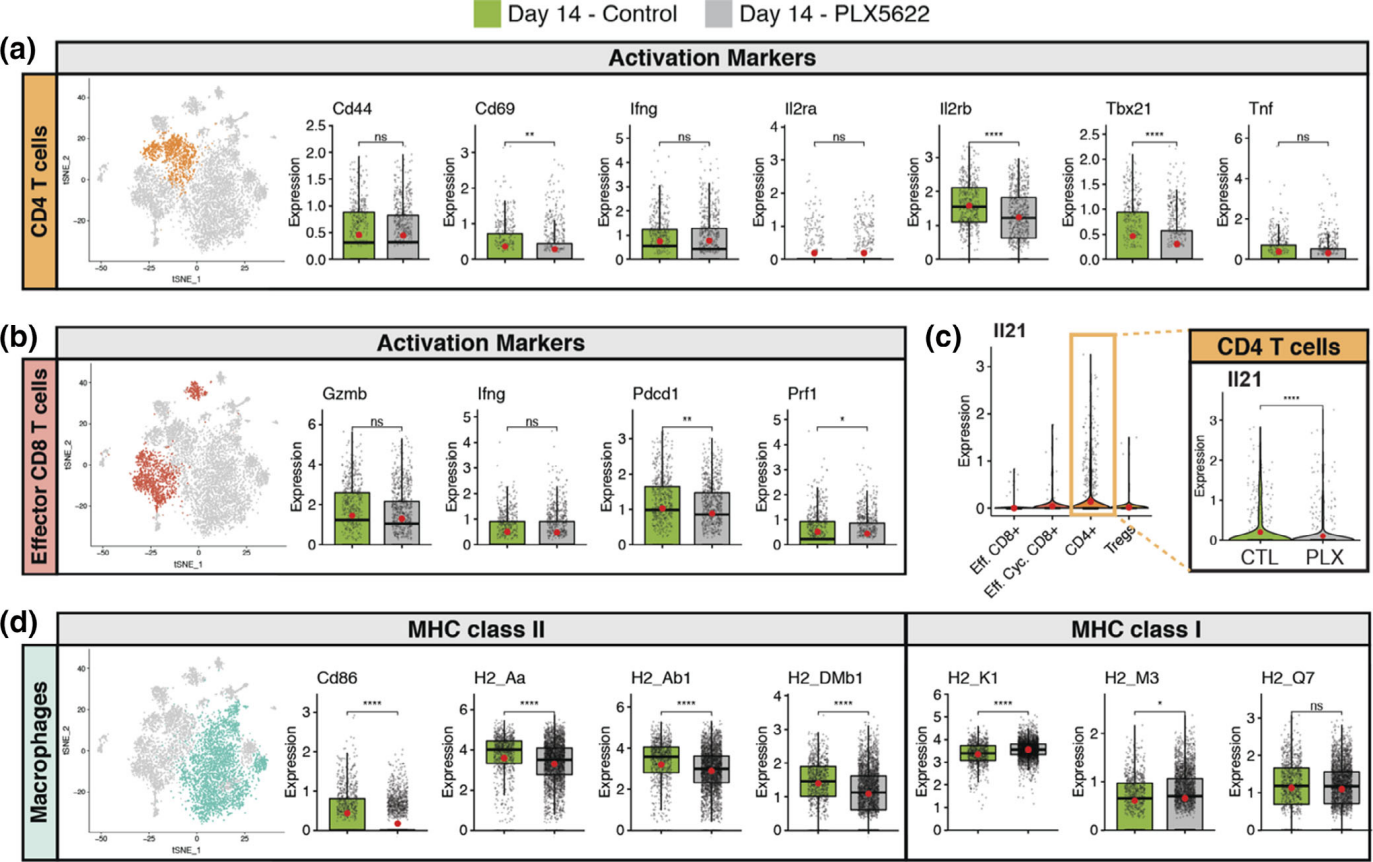
Muted activation profiles of spinal cord CD4+ T cells isolated from PLX5622‐treated mice at day 14 p.i. JHMV‐infected mice treated with either control chow or PLX5622 were sacrificed at day 14 p.i. and CD45+ cells sorted from spinal cords to evaluate mRNA expression profiles via scRNASeq. (a) CD4+ T cells from spinal cords of mice treated with PLX5622‐treated or control mice, comparing expression of transcripts encoding for activation markers *Cd44*, *Cd69*, *Il2ra*, *Il2rb*, *Tbx21*, *Ifng*, and *Tnf*. (b) Expression levels of transcripts encoding effector and activation markers in CD8+ T cells *Prf1*, *Pdcd1*, *Gzmb*, and *Ifng*. (c) Violin plots depicting expression of *Il21* transcripts within T cell populations isolated from the spinal cords of experimental mice; expression is reduced (*p* < .0001) in CD4+ T cells from PLX5622‐treated mice compared to controls. (d) Expression of MHC class II‐associated genes (*H2‐Aa*, *H2‐Ab1*, and *H‐2DMb1*) and co‐stimulatory molecule *Cd86*, as well as MHC class I‐associated genes (*H2‐K1*, *H2‐M3*, and *H2‐Q7*), are shown in macrophages from experimental mice. In these plots, each dot represents a single cell. Normalized expression values were used and random noise was added to show the distribution of data points. The box plot shows interquartile range and the median value (bold horizontal bar). Average expression per sample is represented by the red dot. Wilcoxon test was used for statistical analysis. ns, not significant; *p ≤ .05; ** *p* ≤ .01; ****p ≤ .0001

CNS inflammatory T cells augment demyelination in JHMV‐infected mice presumably through recognition of viral antigens resulting in secretion of cytokines for example, IFN‐γ that activate both resident CNS cells and inflammatory macrophages/myeloid cells to secrete proinflammatory cytokines/chemokines as well as molecules damaging to oligodendrocyte function (Hosking & Lane, 2009; Templeton & Perlman, 2007). By day 14 p.i., we detected no difference in CD4+ T cells in the spinal cords of PLX5622‐treated mice versus controls, though there was an increase (*p* < .05) in CD8+ T cells (Figure S2a) as well as virus‐specific CD4+ (Figure S2b) and CD8+ T cells (Figure S2c) compared with controls. Similar to what we observed at day 7 p.i., we detected reduced expression of *Cd69* transcripts (*p* < .01), *Il2rb* transcripts (*p* < .0001), and *Tbx21* (*p* < .0001) in CD4+ T cells isolated from spinal cords of PLX5622‐treated mice compared to controls (Figure 6a). There were no differences in expression of *Cd44* or cytokines *Ifng* and *Tnf* within CD4+ T cells between experimental groups of mice (Figure 6a). However, *Il21* transcript levels were significantly (*p* < .0001) reduced in CD4+ T cells isolated from PLX5622‐treated mice compared to control mice at this time (Figure 6c). In addition, there was no difference in transcript levels for *Gzmb* or *Ifng* in CD8+ T cells isolated from spinal cords of PLX5622‐treated mice compared with controls (Figure 6b). However, expression of both *Pdcd1* (*p* < .01) and *Prf1* (*p* < .05) were reduced in CD8+ T cells from PLX5622‐treated mice versus controls (Figure 6b). PLX5622‐treatment resulted in reduced expression of *Cd86* (*p* < .0001), *H2_Aa* (*p* < .0001), *H2_Ab1* (*p* < .001), and *H2_DMb1* (*p* < .0001) in spinal cord macrophages compared with control mice (Figure 6d). In contrast, expression of MHC class I‐associated transcripts *H2_K1* (*p* < .0001), H2_M3 (*p* < .05) remained elevated in spinal cord macrophages isolated from PLX5622‐treated mice compared to controls yet there was no difference expression of *H2_Q7* transcripts (Figure 6d).

**FIGURE 7:**
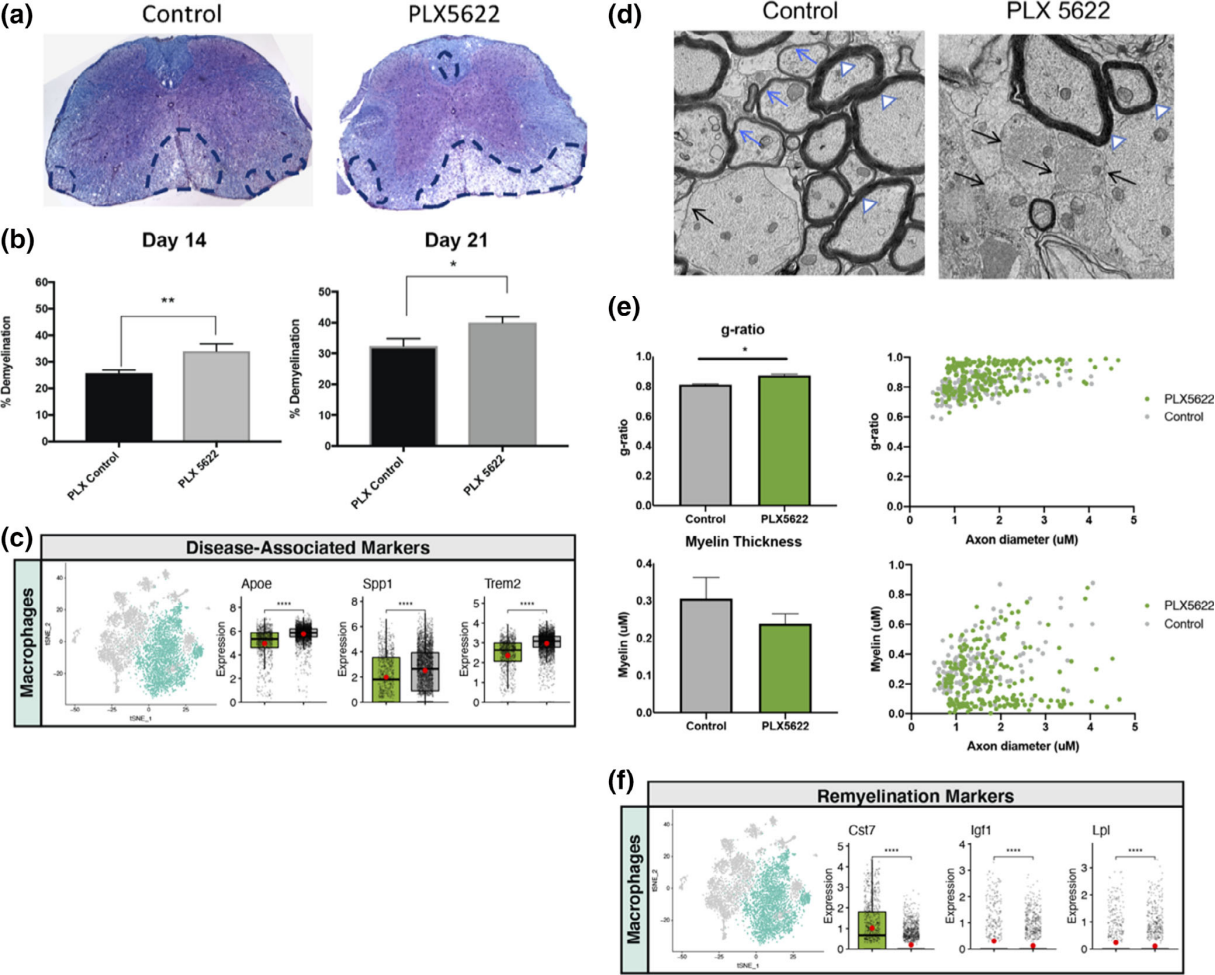
The severity of spinal cord demyelination is increased in PLX5622‐treated mice compared with control mice. (a) Representative images of H&E/LFB‐stained spinal cord sections showing an increase in severity of demyelination (dashed black lines) in JHMV‐infected mice treated with PLX5622 compared with control treated mice at day 14 p.i. (b) Quantification of spinal cord demyelination reveals a significant increase after PLX5622 treatment compared with control‐treated animals (minimum of 12 mice/experimental group) at days 14 and 21 p.i. (c) t‐SNE plots showing increased expression of transcripts encoding disease‐associated factors APOE (*Apoe*), Osteopontin (*Spp1)*, and TREM2 (*Trem2*) in spinal cords of PLX5622‐treated mice compared with controls at day 14 p.i. Normalized expression values were used, and random noise was added to show the distribution of data points. The box plots show interquartile range and the median value (bold horizontal bar). (d) Representative EM images (×1,200) from spinal cords from control and PLX5622‐treated mice showing normal myelinated axons (white arrowheads), demyelinated axons (black arrows), and remyelinated axons (blue arrows) at day 21 p.i. **(**e**)** Calculation of *g*‐ratio of control and PLX5622; scatter plot depicting individual *g*‐ratio's from lateral white matter columns of control (gray) and PLX5622 (green) treated mice as a function of axon diameter. Data in panels b and e are presented as mean ± *SEM*. Average expression value per sample is indicated by the red points. Wilcoxon's test was used for statistical comparisons. (f) t‐SNE plots showing decreased expression of transcripts encoding remyelination‐associated markers Cystatin F (*Cst7*), Insulin growth factor 1 (*Igf1*), and Lipoprotein lipase (*Lpl*) in spinal cords of PLX5622‐treated mice compared with controls at day 14 p.i. *p < .05, *p* < .01, ***p < .001, ****p < .0001

We next assessed how PLX5622 treatment affected the severity of demyelination in JHMV‐infected mice. Using Luxol Fast Blue (LFB) staining of spinal cords from experimental mice, we determined that PLX5622 treatment resulted in an increase in the severity of demyelination at days 14 (*p* < .01) and 21 p.i. (*p* < .05) compared with control mice (Figure 7a,b). These findings indicate that targeting microglia via PLX5622 treatment results in increased demyelination arguing these cells exert a protective role in limiting the severity of white matter pathology in JHMV‐infected mice. We also looked at expression of several genes associated with immune‐mediated demyelinating diseases. Through scRNAseq analysis of CD45 + ‐enriched cells from the spinal cords of JHMV‐infected mice treated with either PLX5622 or control chow, we consistently observed a dramatic increase in transcripts encoding for molecules associated with demyelination including Apoliprotein E (*Apoe*) (Krasemann et al., 2017), Osteopontin (*Spp1*) (Chabas et al., 2001), and Triggering receptor expressed on myeloid cells (*Trem2*) (Krasemann et al., 2017; Ulrich & Holtzman, 2016) in macrophage clusters within the spinal cords of PLX5622‐treated mice compared to controls (Figure 7c). In addition, transcripts encoding proteins associated with remyelination including *Cst7* (Cystatin F), *Igf1* (insulin growth factor 1), and *Lpl* (Lipoprotein lipase) were decreased in spinal cord macrophages within the spinal cords of PLX5622‐treated mice compared to controls (Figure 7f) (Bruce et al., 2018; Durose et al., 2019; Hlavica et al., 2017; Ma et al., 2011; Shimizu et al., 2017; Wlodarczyk et al., 2017; Ye, Li, Richards, DiAugustine, & D'Ercole, 2002). This suggests that microglia may have a role in suppressing expression of molecules associated with demyelination and even promoting expression of those related to remyelination in macrophages. To determine whether remyelination was impacted in response to PLX5622 treatment, EM analysis of spinal cord sections was performed. Assessment of the *g*‐ratio, the ratio of the inner axonal diameter to the total outer fiber diameter, is commonly employed as a structural index of remyelination; lower ratios indicate more extensive myelination (Liu, Keirstead, & Lane, 2001; Moore et al., 2013). High magnification (×1,200) images of the spinal cord ventral funiculus of PLX5622‐treated and control mice were used to evaluate myelinated and demyelinated axons. PLX5622 treatment resulted in a significant (*p* < .05) decrease in remyelination as determined by calculating *g*‐ratio's (0.87 ± 0.004, minimum of 400 axons counted/mouse) compared with control treated mice (0.79 ± 0.003, minimum of 400 axons counted/mouse) (Figure 7d) as well as measuring myelin thickness (Figure 7e).

## 4 DISCUSSION

Viral infection of the central nervous system (CNS) presents unique challenges to the immune system with regard to controlling and eliminating the invading pathogen. The CNS is composed of a variety of highly specialized cells, many of which have limited renewal capacity, that represent potential targets of infection by numerous different viruses (Klein et al., 2019). A significant hurdle encountered by infiltrating antigen‐specific lymphocytes is the elimination of virus from infected cells while limiting the damage that may have long‐term detrimental consequences to the host. Therefore, characterizing the mechanisms involved in how viral infection of the CNS is controlled is an important question. It is becoming increasingly clear that CNS resident cells are critical in host defense through either secretion of anti‐viral cytokines for example, type I interferon (IFN‐I) and/or presentation of antigen within the context of either MHC Classes I or II. Recently, an important role for microglia in aiding in host defense in response to CNS viral infection has been identified (Funk & Klein, 2019; Sanchez et al., 2019a; Seitz et al., 2018; Waltl et al., 2018; Wheeler et al., 2018). Perlman and colleagues (Wheeler et al., 2018) have shown that microglia are required for optimal host defense in response to JHMV infection of the CNS. Targeted depletion of microglia through administration of PLX5622 revealed a role in limiting mortality that was associated with impaired control of JHMV replication. The increase in susceptibility to disease did not appear to be due to altered expression of IFN‐I but more likely a reflection of impaired antigen‐presentation due to muted MHC Class II expression by macrophages infiltrating the CNS of PLX5622‐treated mice and this likely resulted in dampened T cell responses (Wheeler et al., 2018). We believe the increase in expression of IFN‐I response genes in PLX5622‐treated mice most likely reflects the overall increase in viral titers within the brains. Similarly, microglia depletion led to increased mortality in mice infected with WNV associated with diminished activation of APCs and limited reactivation of virus‐specific T cells that led to reduced viral clearance (Funk & Klein, 2019; Seitz et al., 2018). These findings clearly implicate microglia in enhancing optimal host responses following CNS viral infection, in part, by influencing antigen‐presentation that affects virus‐specific T cell responses.

We undertook the present study to better understand how microglia contribute to host defense as well as demyelination and repair following JHMV infection of the CNS using sophisticated molecular, cellular, and histologic approaches. Employing PLX5622 to deplete microglia, we found increased mortality associated with impaired control of CNS viral replication. CSF1R antagonism led to a selective decrease in microglia with sparing of macrophages as well dendritic cells indicating that resident APCs within the CNS were not affected. To better understand the functional contributions of microglia in aiding in host defense, we performed scRNAseq on CD45+ cells enriched from the brains of PLX5622 and control mice at day 7 p.i. Using a verified and unbiased bioinformatics approach (Ekiz et al., 2019), we were able to reliably identify clusters of cells associated with innate immune responses for example, microglia, monocytes, macrophages, NK cells, and neutrophils as well as adaptive responses including T cell subsets and dendritic cells (Figure 2a‐c). Moreover, this approach revealed the heterogeneity of both the innate and adaptive immune cell response by infiltrating cells as well as resident microglia with the identification of different subsets of DCs, macrophages, and T cells. These findings emphasize the complexity of both the innate and adaptive immune responses that occur in response to viral infection of the CNS and raises interesting questions with regard to functional roles for these populations of cells in either defense and/or disease progression. Employing scRNAseq demonstrated that PLX5622‐mediated depletion of microglia did not dramatically alter the presence of the majority of immune cells identified including neutrophils, monocytes, DC and macrophage subsets, as well as Tregs and naïve and memory CD8+ T cells, not to say that these populations are not transcriptionally different. This approach did indicate that PLX5622‐treatment resulted in increased effector CD8+ T cells, CD4+ T cells and, interestingly, B cells. These findings would argue that microglia may not impact either directly or indirectly the synthesis/secretion of proinflammatory cytokines/chemokines by other resident CNS cells for example, astrocytes and/or inflammatory macrophages. Nonetheless, the use of scRNAseq to assess gene expression profiles of CD45+ cells has yielded a tremendous amount of data that we will continue to examine with the goals of elucidating mechanisms not tied to current dogma associated with both anti‐viral host defense mechanisms in response to viral infection of the CNS and viral‐induced demyelination.

Expression of IFN‐I is critical in host defense in response to JHMV infection of the CNS (Athmer et al., 2018; Ireland et al., 2008; Vijay et al., 2017). PLX5622‐mediated targeting of microglia did not affect IFN‐I signaling as GSEA analysis revealed IFN‐α response genes were significantly enriched in both macrophages and dendritic cells within the brains of PLX5622‐treated mice at days 7 compared with controls (Figure 3d,e). These findings may reflect the increase in viral titers within the CNS of PLX5622‐treated mice at these times but also argue that microglia are not solely responsible for production of IFN‐I.

We detected altered T cell responses within the CNS following PLX5622 treatment as determined by both flow cytometry and scRNASeq. There were increased numbers of total CD8+ T cells (*p* < .05) as well as virus‐specific CD8+ T cells within the brains of JHMV‐infected mice treated with PLX5622 compared to controls at day 7 p.i. We also found a trend towards increased total CD4+ T cells and virus‐specific CD4+ T cells in PLX5622‐treated mice compared to controls although these differences were not significant. In terms of T cell activation, we detected differential responses in T cell subsets at day 7 p.i. In CD4+ T cells, there was a reduction in transcripts associated with Th1‐polarized activation *for example*, T‐bet (*Tbx21*) in PLX5622‐treated mice compared to controls although there were no differences in *Ifng* transcripts in CD4+ T cells in experimental groups. PLX5622‐treatment also resulted in reduced expression of CD4+ T cell surface activation markers including CD44, CD69, and components of IL‐2 receptor in PLX5622‐treated mice compared with controls whereas there was an overall increased activation phenotype associated with CD8+ T cells compared with controls. A role for CD4+ T cell‐derived IL‐21 has previously been shown to enhance antiviral CD8+ T cell responses following JHMV infection of the CNS (Phares, DiSano, et al., 2013). While expression of *Il21* transcripts in CD4+ T cells was not affected at day 7 p.i. in PLX5622‐treated mice, expression was significantly reduced by day 14 p.i. This reduction in *Il21* expression in CD4+ T cells in PLX5622‐treated mice may have impacted virus‐specific CD8+ T cell function and partially explain mechanisms associated with impaired control of JHMV replication within the CNS. In addition, the muted activation phenotype by CD4+ T cells was associated with reduced expression of MHC class II by macrophages, but not DCs, at day 7 p.i. in PLX5622‐treated mice, demonstrating a selective effect in response to microglia depletion. This selective effect of reduced MHC class II expression by macrophages in PLX5622‐treated mice was further emphasized in that expression of MHC class I transcripts was increased in macrophages, but not DCs, compared with control mice. Identifying the mechanisms by which microglia augment expression of MHC class II on macrophages will be a focus of ongoing studies.

Our results examining immune cell infiltration and activation in spinal cords of experimental mice at day 14 p.i. through scRNAseq were intriguing as we observed differences in both macrophage and microglial populations compared with the brain at day 7 p.i., regardless of the experimental groups. First, there were both *Ifit +* and *CD40+* macrophages present within the spinal cords of both PLX5622 and control mice, suggesting these cells may be responsible for host defense in response to viral infection (Diamond & Farzan, 2013). It is important to note that both populations of cells were present at a low frequency and there were no dramatic differences between experimental groups, although *CD40+* macrophages were present at a much higher frequency compared to *Ifit +* macrophages. Furthermore, there was a small population of Galectin+ microglia in spinal cords in control mice yet not in PLX5622‐treated mice and previous studies argue for a role for certain isoforms of Galectin in potentially contributing to demyelination in patients with multiple sclerosis (MS) (de Jong et al., 2018) while other isoforms are considered important in driving oligodendrocyte differentiation associated with remyelination (Thomas & Pasquini, 2018). Our findings that spinal cord demyelination was significantly increased in PLX5622‐treated mice supports an emerging role for microglia in restricting the severity of white matter and this is consistent with a recent study from our group indicating that microglia influence the severity of demyelination in JHMV‐infected mice (Brown et al., 2019). While we are currently exploring the molecular and cellular mechanisms by which microglia may modulate the CNS microenvironment in mice persistently infected with JHMV, evidence presented in the current study indicates that PLX5622 treatment resulted in increased expression of transcripts encoding for Osteopontin, APOE, and TREM2 all of which have been implicated in contributing to demyelination (Chabas et al., 2001; Krasemann et al., 2017; Ulrich & Holtzman, 2016). Interestingly, expression of all three transcripts were enriched within macrophage populations suggesting a specific effect by which microglia may suppress expression and limit myelin damage.

Emerging studies have pointed to a protective role for microglia in limiting neuropathology and promoting repair (Baaklini, Rawji, Duncan, Ho, & Plemel, 2019; Lee, Hamanaka, Lo, & Arai, 2019; Lloyd & Miron, 2019). In support of this concept are recent studies from Miron and colleagues (Lloyd & Miron, 2019) showing an important role for microglia in enhancing remyelination in a toxin‐model of demyelination that is aided by microglial death and subsequent microglial repopulation; here, in this study, the absence of microglia prevents their further death and repopulation and is associated with increased white matter damage. Although mechanisms by which microglia may support remyelination have not been completely defined, it is though that these cells aid in clearance of myelin debris and/or secrete growth factors/cytokines that influence maturation of oligodendrocyte progenitor cells (OPCs) into mature myelin‐producing oligodendrocytes. What is also clear is that microglia are heterogenous in terms of transcriptome and protein expression which is likely regulated during disease and this would influence the role of these cells in enhancing or muting disease progression and potential repair. In support of a protective role for microglia in restricting neuropathology and promoting repair is our data demonstrating that PLX5622 treatment of JHMV‐infected mice results in an increase in white matter damage associated with impaired remyelination (Figure 7a,b,d, and e). In addition, scRNASeq also shows reduced expression of genes encoding proteins previously associated with remyelination including Cystatin F (Durose et al., 2019; Ma et al., 2011; Shimizu et al., 2017), Insulin growth factor 1 (IGF1) (Hlavica et al., 2017; Wlodarczyk et al., 2017; Ye et al., 2002) and Lipoprotein lipase (Bruce et al., 2018) within macrophages isolated from the spinal cords of PLX5622‐treated mice (Figure 7f). These findings further support the notion that microglia may either directly or indirectly influence remyelination within the spinal cord by contributing to controlling expression of genes encoding proteins that regulate OPC maturation. We are currently pursuing the functional contributions of Cystatin F, IGF1, and Lipoprotein lipase in contributing to remyelination in JHMV‐infected mice.

We would caution that although demyelination was worsened and remyelination was impaired when microglia are depleted, this may reflect the model employed that is, targeted depletion of microglia prior to JHMV infection of the CNS which may lead to increased neuropathology through altered macrophage biology and/or resident cells of the CNS including astrocytes and oligodendrocytes. We are currently determining if microglia depletion in mice persistently infected with JHMV in which demyelination is established affects either neuropathology and/or viral recrudescence as this is a more clinically‐relevant question. Similarly, we are also determining if there is efficient repopulation of microglia in mice persistently infected with JHMV upon removal of PLX5622 treatment. Another important area we are pursuing relates to whether depletion of microglia in mice with established demyelination impacts remyelination.

## CONFLICT OF INTEREST

The authors declare no competing financial interests.

## Supporting information


**Supplemental Figure 1** **PLX5622 treatment influences T cell infiltration and activation state within the CNS of JHMV‐infected mice (A)** Representative flow cytometric plots showing CD4^+^ and CD8^+^ T cells infiltrating into the brains of JHMV‐infected mice treated with either PLX5622 or control at day 7 p.i. Quantification of flow data indicates increased infiltration of CD4+ T cells and CD8+ T cells (*p* < 0.05) increase in brains of PLX5622‐treated mice compared to controls, (n = 6/group). Representative tetramer staining revealed increased infiltration of (**B**) virus‐specific CD4+ T cells and (**C**) virus‐specific CD8+ T cells within the brains of PLX5622‐treated mice compared to controls at day 7 p.i., (n = 6/group). Data are derived from 2 independent experiments and presented as mean ± SEM. (*p ≤ 0.05; ** *p* ≤ 0.01).

**Supplemental Figure 2** **PLX5622 modulates T cell infiltration into the spinal cord.** (**A)** Flow analysis of spinal cords at day 14 p.i. from JHMV‐infected mice indicate an increase in CD4+ T cells and CD8+ T cells (*p* < 0.05) in PLX5622 compared to controls. PLX5622 treatment results in increased spinal cord infiltration of (**B**) virus‐specific CD4+ T cells (*p* < 0.05) and (**C**) virus‐specific CD8+ T cells (*p* < 0.01), (n = 5/group). Data are derived from 2 independent experiments and presented as mean ± SEM. (*p ≤ 0.05; ** *p* ≤ 0.01).

## Data Availability

The data that support the findings of this study are available from the corresponding author upon reasonable request.
