# Supplementary figures and images for: Microglia influence host defense, disease, and repair following murine coronavirus infection of the central nervous system
Source: Glia. 2020 May 25;68(11):2345–60. doi: 10.1002/glia.23844 (PMC7280614; doi:10.1002/glia.23844)

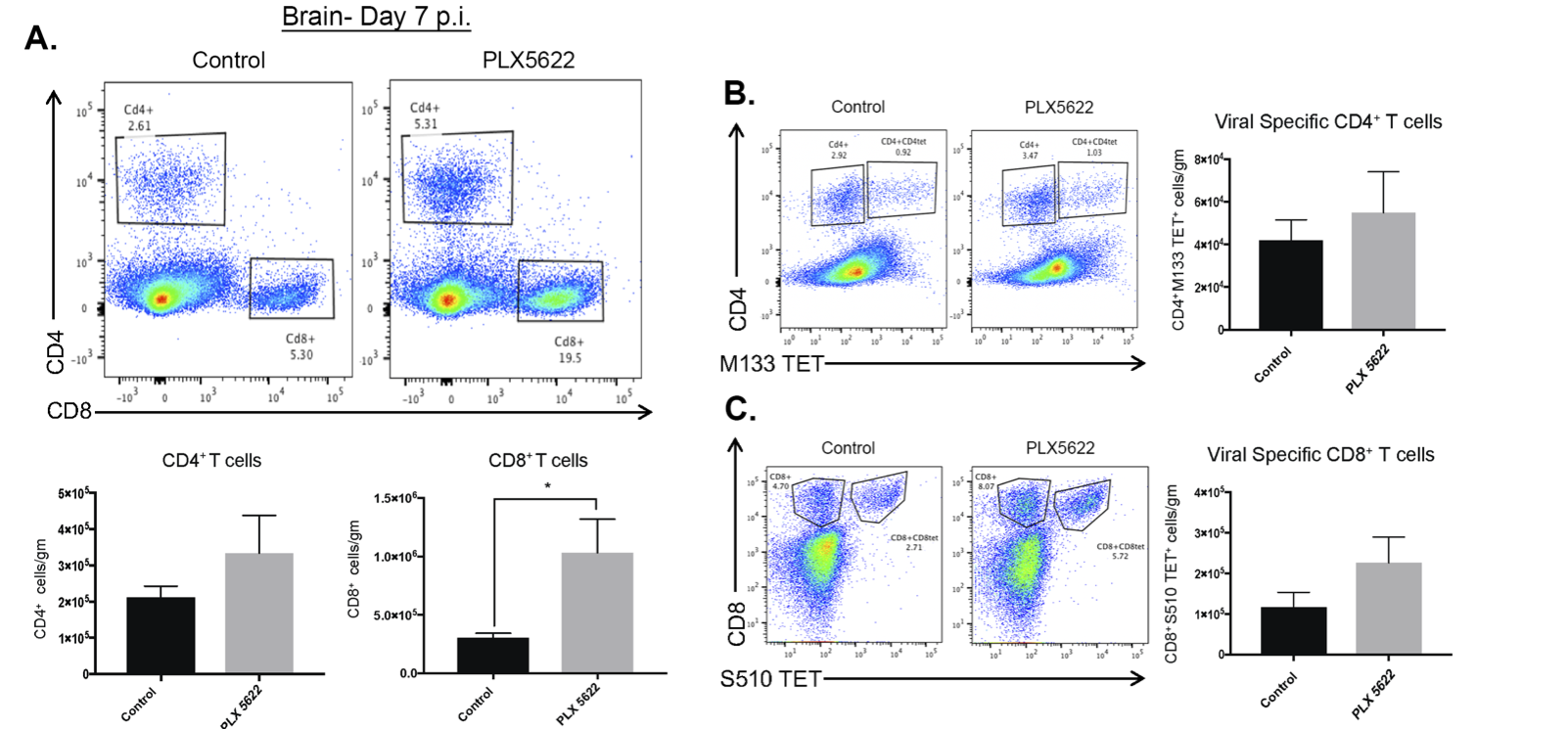

Supplement: Supplementary file 1 — Supplemental Figure 1 PLX5622 treatment influences T cell infiltration and activation state within the CNS of JHMV‐infected mice (A) Representative flow cytometric plots showing CD4+ and CD8+ T cells infiltrating into the brains of JHMV‐infected mice treated with either PLX5622 or control at day 7 p.i. Quantification of flow data indicates increased infiltration of CD4+ T cells and CD8+ T cells (p < 0.05) increase in brains of PLX5622‐treated mice compared to controls, (n = 6/group). Representative tetramer staining revealed increased infiltration of (B) virus‐specific CD4+ T cells and (C) virus‐specific CD8+ T cells within the brains of PLX5622‐treated mice compared to controls at day 7 p.i., (n = 6/group). Data are derived from 2 independent experiments and presented as mean ± SEM. (*p ≤ 0.05; ** p ≤ 0.01). [file GLIA-68-2345-s002.tif]

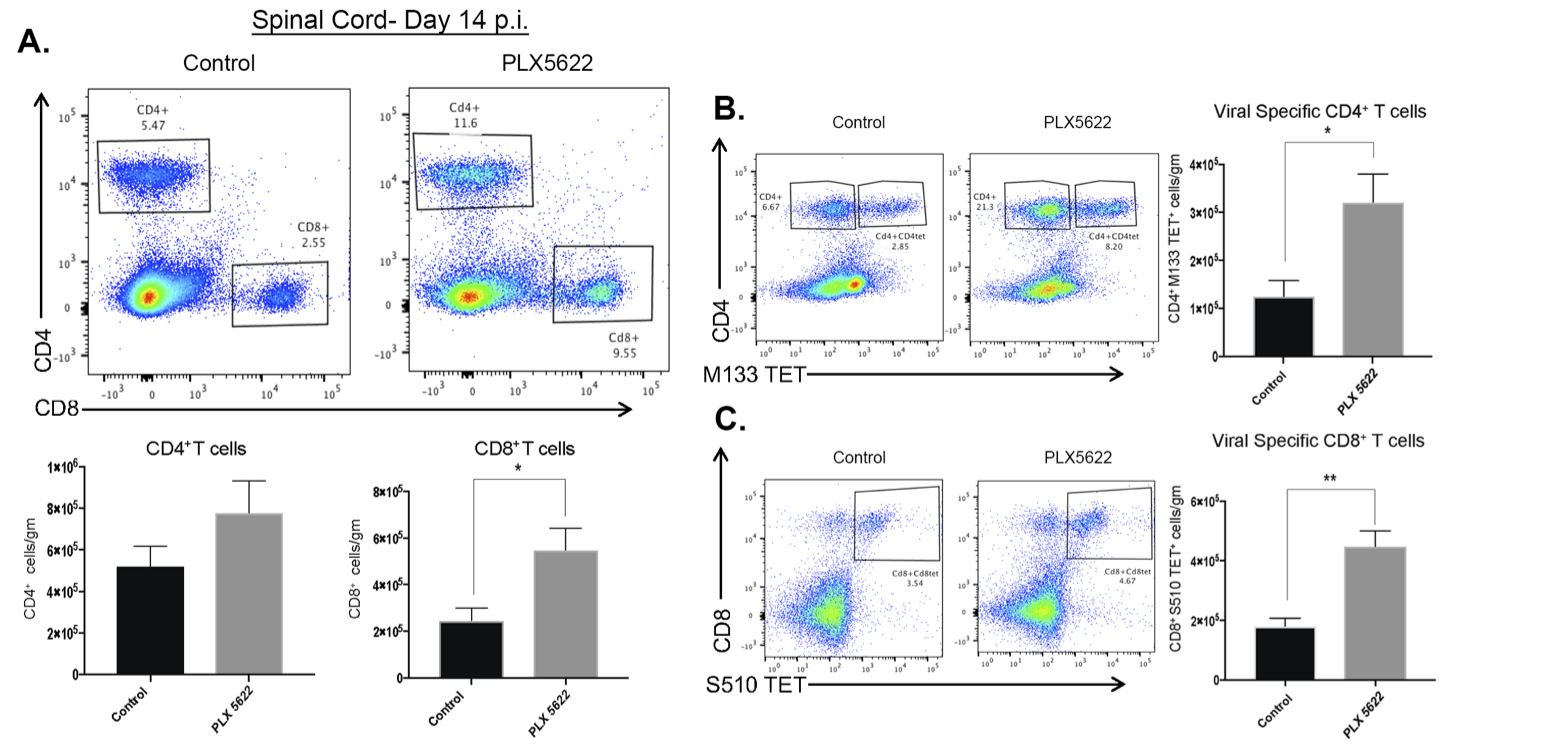

Supplement: Supplementary file 2 — Supplemental Figure 2 PLX5622 modulates T cell infiltration into the spinal cord. (A) Flow analysis of spinal cords at day 14 p.i. from JHMV‐infected mice indicate an increase in CD4+ T cells and CD8+ T cells (p < 0.05) in PLX5622 compared to controls. PLX5622 treatment results in increased spinal cord infiltration of (B) virus‐specific CD4+ T cells (p < 0.05) and (C) virus‐specific CD8+ T cells (p < 0.01), (n = 5/group). Data are derived from 2 independent experiments and presented as mean ± SEM. (*p ≤ 0.05; ** p ≤ 0.01). [file GLIA-68-2345-s001.tif]
